# Impact of vector biology research on old and emerging neglected tropical diseases

**DOI:** 10.1371/journal.pntd.0006365

**Published:** 2018-05-31

**Authors:** Jesus G. Valenzuela, Serap Aksoy

**Affiliations:** 1 Vector Molecular Biology Section, Laboratory of Malaria and Vector Research, National Institutes of Allergy and Infectious Diseases, National Institutes of Health, Bethesda, Maryland, United States of America; 2 Department of Epidemiology of Microbial Diseases, Yale School of Public Health, New Haven, Connecticut, United States of America; University of Washington, UNITED STATES

## Overview

Vector-borne diseases (VBDs) represent a great proportion of the neglected tropical diseases (NTDs) in tropical regions of the world, where they disproportionately affect the poorest and most disadvantaged populations. That said, in recent years, the expansion of vectors from the tropics has placed many more people living in the temperate regions of the world at risk of contracting VBDs. The expansion of VBDs is occurring at a time when unprecedented discoveries are being made in vector biology in the areas of genetics, genomics, and physiology. With the goal of highlighting the significance of VBDs in the new century and emphasizing the role of basic vector research in the development of future disease control methods and for improving upon the existing approaches, we have advanced a call for papers that focused on vector research as part of our 10th anniversary celebration. It is our hope that these new discoveries in vector biology research will pave the way for translational methods to combat the old foes in the new climate.

According to the World Health Organization (WHO), nearly half of the world’s population is infected with at least one type of vector-borne pathogen [1]. Among these pathogens are the causative agents of NTDs, including African sleeping sickness, Chagas disease, leishmaniasis, lymphatic filariasis, schistosomiasis, onchocerciasis, plague, and typhus as well as dengue, Zika fever, chikungunya, and Crimean–Congo hemorrhagic fever. Most of the vectors that transmit these disease agents are bloodsucking arthropods, which ingest disease-producing microorganisms during a blood meal from an infected host (human or animal) and later inject it into a new host during their subsequent blood meal. Among these arthropods are primarily mosquitoes but also tsetse flies, triatomine bugs, sand flies, snails, black flies, fleas, and ticks. The burden of VBDs has been the highest in the tropical regions of the world, where they disproportionately affect the poorest and most disadvantaged populations. That said, increased global travel in the last several decades and the recent expansion of vectors from the tropics has placed many more people living in the temperate regions of the world at risk of contracting VBDs.

By the late 19th century, most of the major VBD agents were identified, including the malaria parasite, for which Charles Louis Alphonse Laveran, a French army surgeon stationed in Constantine, Algeria, was awarded the Nobel Prize in 1907. The transmission cycle of malaria by mosquitoes was also discovered, for which Ronald Ross, a British officer in the Indian Medical Service, was awarded the Nobel Prize in 1902. Accounts of sleeping sickness were noted by ships’ doctors and medical officers who worked for slave-trading companies in the 17th and 18th centuries. David Bruce, the Scottish microbiologist, discovered *Trypanosoma brucei* as the cause of cattle trypanosomiasis (cattle nagana) in 1895, and the British colonial surgeon Robert Michael Forde was the first to observe trypanosomes in the human blood in 1901. Both David Bruce and the German military surgeon Friedrich Karl Kleine provided conclusive evidence showing the cyclical transmission of *T*. *brucei* in tsetse flies at the beginning of 1900 (for a review, see [2]). Discovery of the pathogen transmission cycles, as well as applications of insecticides to reduce vector populations, enabled the control of these diseases for much of the 20th century.

Recently, old foes have emerged or re-emerged and become a topic of growing importance in public health and in political and scientific agendas [3]. Besides the resurgence of malaria as a result of pesticide and drug resistance, a plethora of arboviral diseases have emerged and re-emerged, including dengue fever in Asia and the Americas; chikungunya fever in Asia, Africa, and Europe [4–7]; Zika fever in Asia and the Americas; yellow fever in Africa and the Americas; the tick-borne Powassan virus in the United States of America; and Crimean–Congo hemorrhagic fever in Europe. Several factors are contributing to the re-emergence of VBDs. On the one hand, the spread of resistance to drugs in pathogens has become a major obstacle for the effective treatment of some VBDs [8–10], and the emergence of new strains of arboviruses has created new challenges for healthcare systems. The Zika virus has received considerable attention over the past two years as a major public health crisis, particularly in the Americas [11]. On the other hand, an increase in insecticide resistance is threatening the sustainability of vector control programs in several tropical regions [12, 13]. Additionally, the expansion of vector populations due to climate change is becoming a growing concern in temperate countries, where control programs have been discontinued for almost 50 years [14].

The expansion of VBDs is occurring at a time when unprecedented discoveries are being made in vector biology in the areas of genetics, genomics, and physiology. It is our hope that these new discoveries on basic and applied research in vector biology will pave the way for translational vector and pathogen control methods to combat the old foes in the new climate. With the goal of highlighting the significance of VBDs in the new century and emphasizing the role of basic vector research in the development of future disease control methods, we have advanced a call for papers that focused on VBDs as part of our 10th anniversary celebration. We have gathered papers with an emphasis on different aspects of vector control, including components that affect mosquito physiological functions, new portable protective mosquito traps, novel mosquito repellents, and a low-tech insecticide release device. A good number of papers in this collection address the area of vector–pathogen interactions, including viral co-infections in mosquitoes, a parasite transporting viruses in the insect gut, how the Zika virus alters mosquito RNA interference (RNAi) response, and how the enzyme catalase protects mosquitoes from oxidative stress and helps dengue virus survival in the insect gut. We have also received a number of papers on triatomine-transmitted Chagas disease, which highlights the increasing significance of this disease burden in South America. These papers highlight the relevance of basic research on vector–pathogen interactions to find new venues to control transmission of VBDs. Many of the articles highlight the complexity of vector–pathogen–host interactions, which can serve as novel points of interference for control, including the role of pathogen-produced exosomes at the bite site, the role of vector saliva pre-exposure in disease, and viral modification of host gene expression.

Research in vector microbiota continues to attract attention as commensal and symbiotic microbes harbored by insects can influence various disease transmission traits, including an effect on the gut barriers to prevent malaria infection in mosquitoes. In the last decade, the *Wolbachia* endosymbionts have gained attention because of the various host reproductive modifications they induce in order to enable their transmission (and that of the infected vector) through natural populations. Mosquitoes infected with a novel strain of *Wolbachia* have also been shown to resist transmission of dengue [15], chikungunya [16], and Zika virus [17]. One translational application of this discovery, *Wolbachia* endosymbiont–mediated pathogen interference, is currently being tested in trials against dengue in Southeast Asia and in implementation trials against Zika virus disease in Colombia and Brazil. Our collection highlights several papers that investigate *Wolbachia* effects on mosquito disease transmission traits and model the success of these applications.

Improved knowledge of climate change effects and vector habitats, coupled with mathematical modeling efforts, can predict transmission dynamics and enhance the efficacy of ongoing or future control efforts in the field. The diversity of vectors and diseases presented in the Vector Biology collection emphasizes the growing significance of this area and these research investigations towards effective control. The ultimate goal is for the accumulating knowledge on vector–pathogen dynamics to inform and guide public health decisions for optimal outcomes. Several of the papers in this collection begin to make the link between basic science- and field-based discoveries and policy decisions. Additional papers, which are currently under review in our system, will be added to the collection.

VBDs represent a great proportion of the NTDs. The high-quality vector biology research presented by various research groups from around the globe that includes basic research, field work, modeling, and translational research demonstrates the commitment to find new avenues towards the control of these NTDs. Importantly, this collection reinforces the commitment of our editorial board to continue publishing high-quality and impactful research in vector biology as they impact control of VBDs.
